# CASE REPORT Sternal Chondrosarcoma After Sternotomy for Coronary Artery Bypass Grafting

**Published:** 2013-01-24

**Authors:** Vijay A. Singh, Juan Abreu, Kimberly Bowman, Masood A. Shariff, Joseph T. McGinn

**Affiliations:** ^a^Cardiothoracic Surgery Department; ^b^Department of Surgery, Staten Island University Hospital, Staten Island, NY

## Abstract

**Objective:** Primary bony tumors of the chest wall are usually benign and most commonly located in the ribs or sternum. Chondrosarcoma is regarded as one of the most frequent primary malignancies of the chest wall and its incidence after a sternotomy for a cardiac procedure is extremely rare. We present a case of sternal chondrosarcoma. **Methods:** The patient presented with a sternal mass 4 years after undergoing coronary artery bypass grafting for ischemic coronary artery disease. The mass originally emanated from the upper portion of the patients’ sternum and then rapidly enlarged to include the anterior aspects of his neck. Radiologic imaging studies were undertaken: computed tomographic scan and magnetic resonance imaging, with surgical intervention for excision. **Results:** Computed tomographic scan and magnetic resonance imaging established an 8.4 × 6.2 × 8.6 cm^3^ complex solid tissue mass within the lower neck arising from the sternal manubrium, with extensive bone destruction. Computed tomography-guided biopsy showed cells of uncertain significance. Surgical excision was performed and the mass was diagnosed as a grade II chondrosarcoma. **Discussion:** Primary sarcomas of the sternum though uncommon are potentially curable with wide surgical excision. Success depends on tumor histologic type and grade, which dictate recurrence.

Primary bony tumors of the chest wall are usually benign and most commonly located in the ribs or sternum.^1^ Categorization of the lesions preoperatively may be difficult. Thus, optimal management includes wide local excision until proven benign.^1^^,^^2^ Although chondrosarcoma is regarded as one of the most frequent primary malignancies of the chest wall and sternum its relative occurrence is rare. It has an incidence of about 0.5 cases per million per year.^3^ Sternal chondrosarcoma after sternotomy in a patient with no previous history of malignancy has not been reported in the literature. Differential diagnosis of an anterior chest wall mass includes granular cell tumor, enchondroma, fibroma, osteosarcoma, and pseudoaneurysms among others.^4^ This article presents a case of a sternal chondrosarcoma presenting 4 years after a sternotomy. Its imaging, pathological appearance, and surgical treatment are discussed.

## METHOD/CASE PRESENTATION

**Figure 1:**
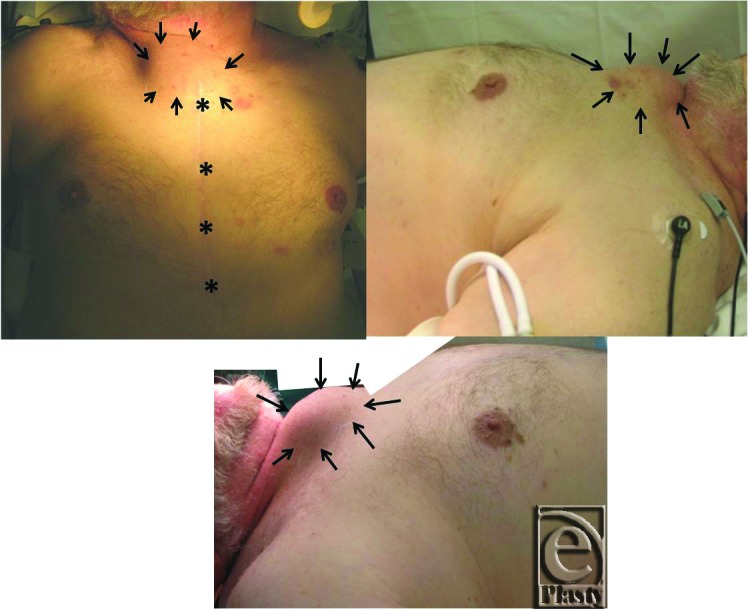
Overgrowth of the tissue can be seen delineated by black arrows. Frontal view shows the previous coronary artery bypass grafting scar (*, previous sternotomy scar; arrows, mass outlined).

A 77-year-old man, ex-smoker, with a medical history remarkable for coronary artery disease status post quadruple coronary artery bypass grafting 5 years ago (2008), diabetes, hypertension, congestive heart failure, atrial fibrillation, and abdominal aortic aneurysm repair with stent placement also in 2008. The patient is a Vietnam War veteran and was in the war zone where Agent Orange was utilized. He presented to the cardiothoracic surgery office for outpatient evaluation after noticing an expanding upper sternal mass, which he first observed 3 months prior (Fig 1). The patient had no complaints of difficulty breathing, chest pain, or coughing. Radiologic survey utilizing computed tomographic (CT) scan and magnetic resonance imaging was conducted to further evaluate the mass, followed by surgical intervention and pathological analysis.

## RESULTS

**Figure 2:**
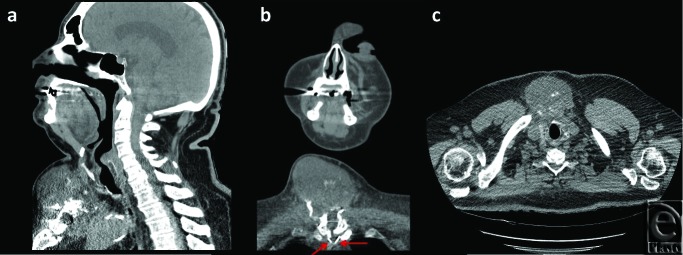
Computed tomographic scan of the soft tissue in sagittal (*a*), frontal (*b*), and transverse (*c*) planes of the granulation arising from the upper sternum. Sternal wires can be seen (*red arrows*) on the frontal view. The granulation tissue is seen extending anteriorly and posteriorly on the sagittal and transverse planes.

**Figure 3:**
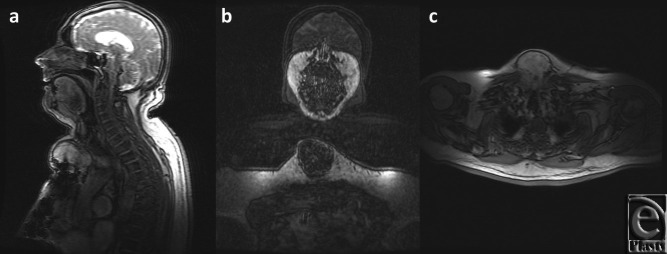
Magnetic resonance images in sagittal (*a*), frontal (*b*), and transverse (*c*) planes of the granulation arising from the upper sternum. The granulation tissue is seen extending anteriorly and posteriorly on the sagittal and transverse planes.

A thorough work-up ensued yielding further details of the mass, as noted on the CT scan (Fig 2) and magnetic resonance imaging (Fig 3), which established this as a 8.4 × 6.2 × 8.6 cm^3^ complex solid tissue mass within the lower neck arising from the sternal manubrium, with extensive bone destruction. Because of the clinical and radiological appearance, a provisional diagnosis of a sternal granuloma was made.

**Figure 4:**
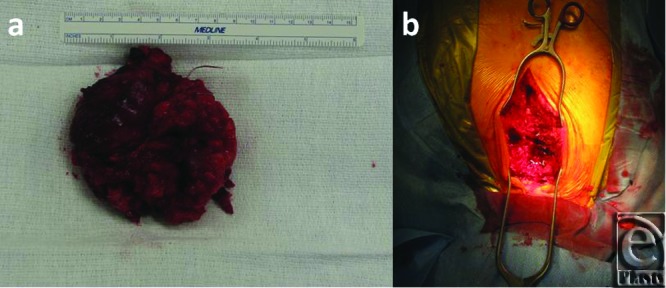
(*a*) Gross mass dissected from the anterior side of the sternum measuring 9 × 9 cm^2^. (*b*) Surgical field.

The CT-guided biopsy showed cells of uncertain significance, and the aspirate demonstrated no abnormal growth. The patient was then scheduled for an elective excision of the mass. During the procedure, an 8.5 × 8.5 × 2.5 cm^3^ mass arising from the anterior portion of the sternal manubrium, extending to the neck and locally invading the skeletal muscle was partially resected (Fig 4). A complete excision could not be performed because of the high-risk location of some parts of mass as well as the patients overall suboptimal health condition. A conclusive diagnosis could not be reached on frozen section at the time of surgery. Therefore, the resection entailed removing all visible aspects of the mass.

Pathologic evaluation diagnosed a Grade II chondrosarcoma. Histopathological findings revealed a cartilaginous neoplasm with some areas of bone infiltration, mixed cellularity, and spindled cytomorphology in a background of myxoid matrix. The patient's overall comorbidities obscure the next step of the process. Nevertheless, he is being prepared to undergo a sternectomy with pectoralis flap closure.

## DISCUSSION

Chondrosarcoma accounts for 20% of the primary tumors of the thoracic wall, 20% of these arise from the sternum. Formed mostly by chondrocytes and a cartilage matrix, chondrosarcomas are mainly found in the pelvis, chest wall, and proximal long bones.^5^ Its peak incidence occurs between the ages 50 to 70 years of age.^6^ Most tumors are primary, but they have also been reported in association with trauma, radiotherapy, chromosomal mutations (such as, neurofibromatosis, bilateral Werner syndrome, and retinoblastoma), fibrous dysplasia, and Paget's disease.^5^^,^^7^^-^10

Primary sarcomas of the sternum though uncommon are potentially curable with wide surgical excision. Using rigid prostheses to repair the skeletal defects, the surgical complication rates are low. Overall survival and tumor behavior after complete surgical resection is related to the histologic type and grade. The latter takes into consideration the cell density, nuclear size, and degree of nuclear staining (hyperchromasia). According to the Evans classification in a scale that can range from Grade I (well differentiated) to Grade III (poorly differentiated), grade is the most critical single predictor of recurrence and metastasis.^7^ Thus, the microscopic examination following resection is crucial for determining the most appropriate management.

Wide resection of malignant chest wall tumors includes the affected ribs with at least a 4 ± 5 cm free margin proximally and distally to the tumor, portions of the ribs immediately above and below the tumor, the adjacent muscles, and the underlying pleura. Any other tissue adherent to the tumor is also excised. For sternal chest wall tumors, the size and location of the tumor determines the extent of the resection. Lesions at the upper third of the sternum necessitate resection of the manubrium and most of the sternal body as well as the medial ends of the clavicles, the adjacent sternocostal cartilages, and subtotal sternectomy. Lesions at the middle third of the sternum are treated with resection of the sternal body, with preservation of the manubrium and the xiphoid process.^1^

Of note, potential complications include multiple myeloma, infection, need for tracheostomy, additional chest wall reconstruction, and even death.^1^^,^^11^^,^^12^ Success depends on tumor histologic type and grade, which dictate recurrence. Patients with low-grade chondrosarcoma and no recurrence can have up to 80% survival after more than 5 years.^1^^,^^11^^,^^12^

Our case demonstrates a Grade II chondrosarcoma, which occurred 4 years after a sternotomy for a coronary artery bypass grafting. The exact genesis of this tumor is not known. Being that this patient was a Vietnam War veteran, the association with Agent Orange (2,3,7,8-tetrachlorodibenzo-*p*-dioxin) cannot be discounted. Agent Orange is the code name for one of the herbicides and defoliants used by the United States military as part of its extensive herbicidal warfare program during the Vietnam War. A number of different types of cancers found in Vietnam War veterans have been loosely attributed to the use of Agent Orange.^13^^,^^14^ However, there is no current demonstrable link between chondrosarcoma and Agent Orange.

This case reveals an interesting scenario where a malignant tumor formed after a sternotomy. Usually, when a large mass is found after sternotomy, the differential diagnosis mainly includes infection, granulomas, or pseudoaneurysms. This article proves that the diagnosis of chondrosarcoma should be included in that differential.
